# Supplementary figures and images for: Gp35/50 mucin molecules of Trypanosoma cruzi metacyclic forms that mediate host cell invasion interact with annexin A2
Source: PLoS Negl Trop Dis. 2022 Oct 3;16(10):e0010788. doi: 10.1371/journal.pntd.0010788 (PMC9529151; doi:10.1371/journal.pntd.0010788)

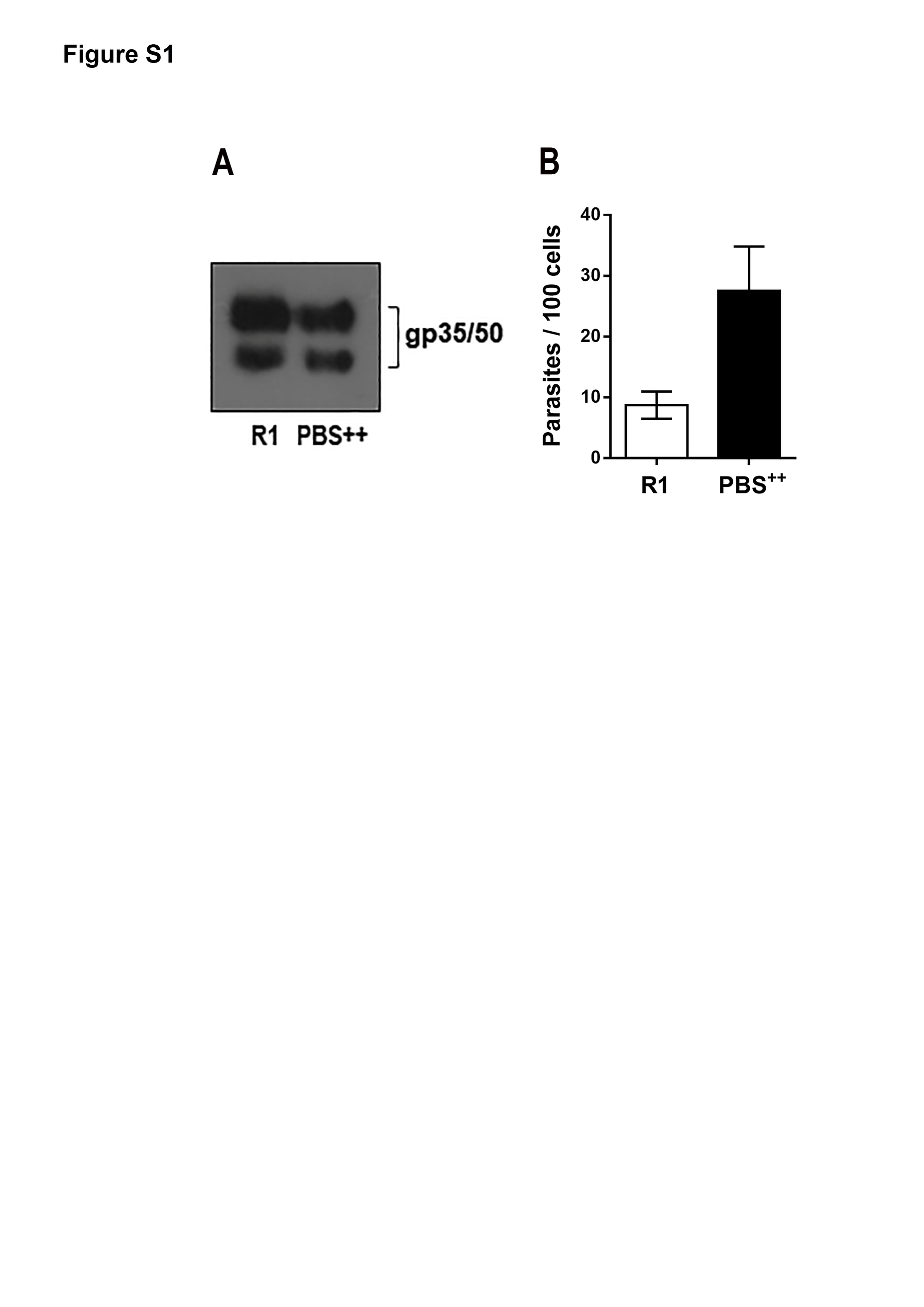

Supplement: S1 Fig — (A) Conditioned medium from parasites, incubated for 30 min in RPMI medium containing 1% serum (R1) or in PBS++, was analyzed by western blotting using mAb 10D8. Note the higher mucin levels in R1. (B) HeLa cells were incubated for 1 h with MT in R1 or PBS++, and the number of intracellular parasites was quantified. Values are the means ± five independent assays. (TIF) [file pntd.0010788.s001.tif]

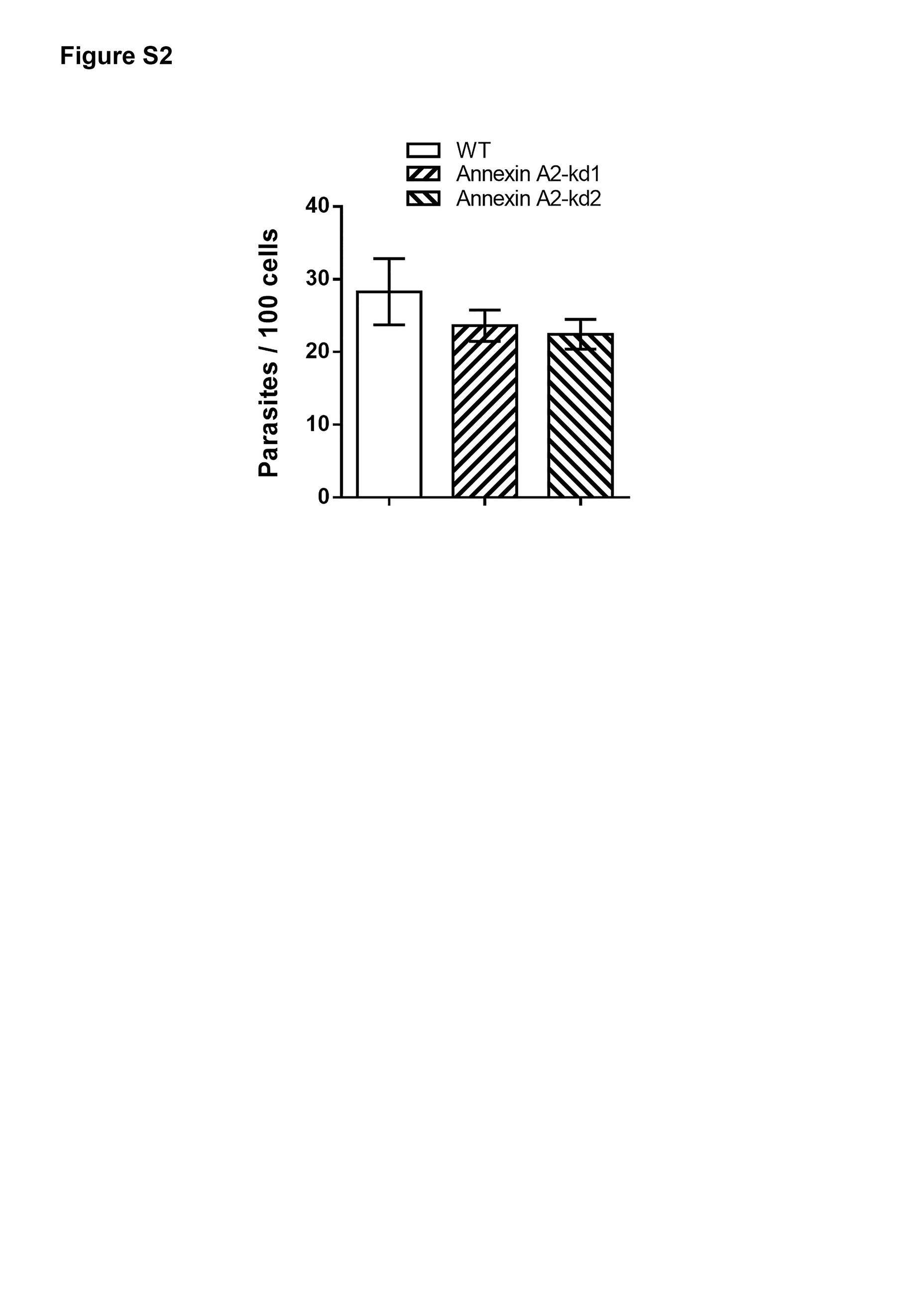

Supplement: S2 Fig — HeLa cells depleted in annexin A2 and WT cells were incubated for 1 h with CL strain MT. The amounts of intracellular parasites are shown as means ± SD of three independent assays performed in duplicate. (TIF) [file pntd.0010788.s002.tif]

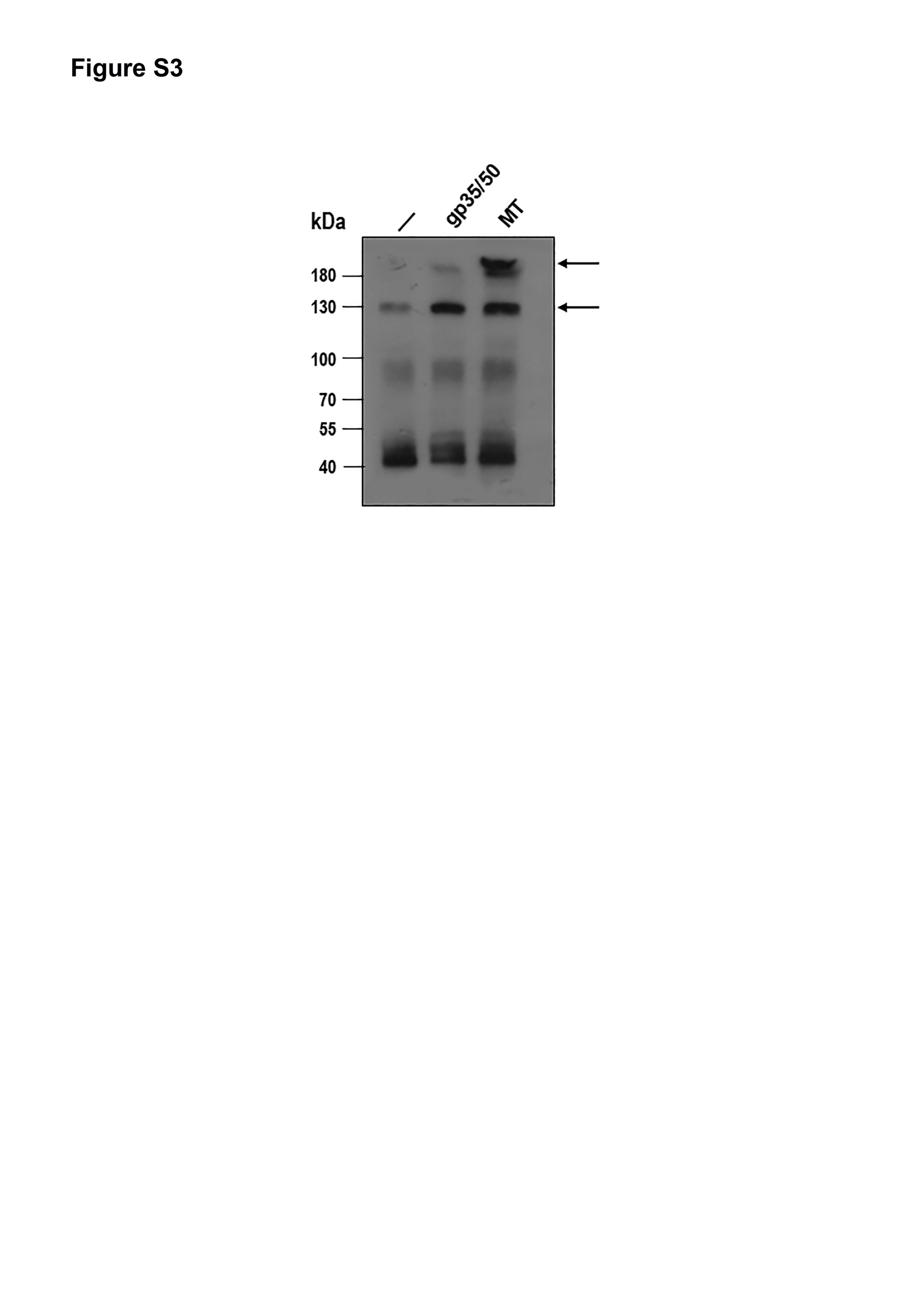

Supplement: S3 Fig — HeLa cells were incubated for 30 min in absence or in the presence of MT or MUC-G at 40 μg/ml. After washings, the cell extracts were analyzed by western blotting, using antibody directed to phosphorylated tyrosine proteins. Note the increased phosphorylation levels of protein bands in cells that interacted with MT or with MUC-G (black arrows). (TIF) [file pntd.0010788.s003.tif]

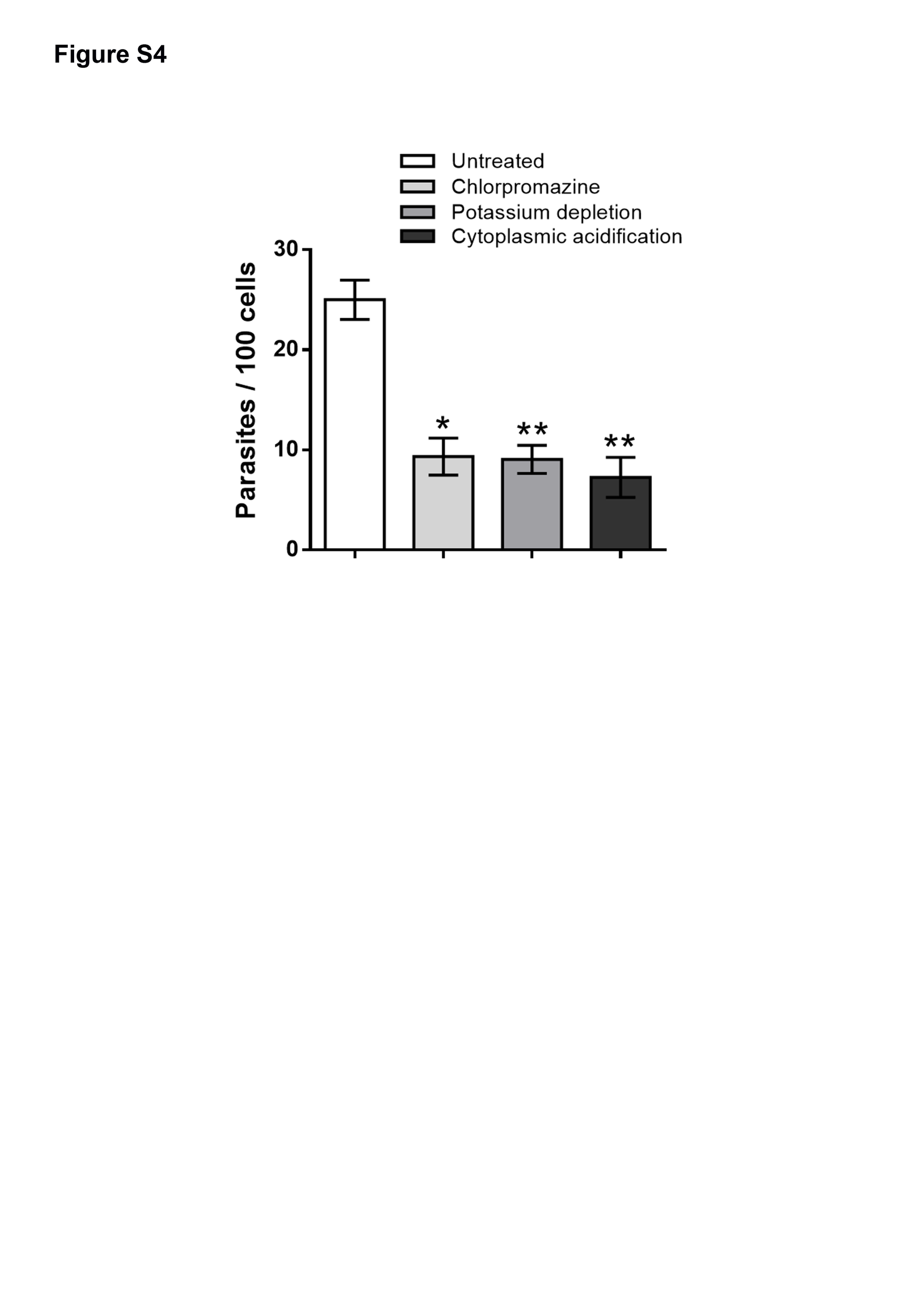

Supplement: S4 Fig — HeLa cells were subjected to chlorpromazine treatment, intracellular K+ depletion or cytoplasmic acidification, and then incubated with MT for 1 h, followed by internalized parasite quantification. Values are the means ± three independent assays performed in duplicate. MT invasion was significantly reduced in cells subjected to diverse procedures (*P<0.001, **P<0.0005). (TIF) [file pntd.0010788.s004.tif]
