# Supplementary material for: Gp35/50 mucin molecules of Trypanosoma cruzi metacyclic forms that mediate host cell invasion interact with annexin A2
Source: PLoS Negl Trop Dis. 2022 Oct 3;16(10):e0010788. doi: 10.1371/journal.pntd.0010788 (PMC9529151; doi:10.1371/journal.pntd.0010788)
Supplement: Attachment — Submitted filename: Response to reviewers.docx [file pntd.0010788.s005.docx]

**Response to reviewers**

**Reviewer 1**

**1.**  The introduction would benefit from some further explanation of what genes encode gp35/50 and gp82 and how that relates to the genomes/transcriptomes/proteomes of G and CLBR strains, or an indication of where there are gaps in this kind of knowledge. Presumably these are multicopy genes, so is it known how many copies are in each strain? For example, it is not clear whether the G strain has gp82 genes or not, or if it has them then are they expressed (in trypos?) or are they very divergent

sequences vs CLBR or have different glycosylation function. Likewise for gp35/50 genes in CLBR.

**R.** **As recommended, more detailed information on gp82 and gp35/50 is provided in the Introduction of the revised manuscript and, accordingly, additional references were incorporated. Gp82 and gp35/50 are structurally quite distinct glycoproteins. Exclusively expressed in MT (Teixeira & Yoshida, 1986), gp82 is encoded by a multigene family (Araya et al., 1994). It contains N-linked oligosaccharides (Ramirez et al. 1993), but the carbohydrate portion of the molecule is irrelevant for host cell adhesion or invasion (Manque et al., 2000). The identity of amino acid sequences of gp82, as deduced from cDNA clones of G and CL strains, is 97.9%** **and, as regards the cell binding domain, the identity is 100% (Yoshida, 2006). Gp35/50 molecules are expressed both in MT and epimastigotes (Yoshida et al., 1989). They are encoded by a multigene family and are highly glycosylated proteins rich in threonine, with a unique type of glycosylation consisting of several sialylated *O*-glycans linked to the protein backbone via *N*-acetylglucosamine residues (Schenkman et al. 1993, Acosta et al., 2001). As to the diversity of gp35/50 mucins in G and CL strain, it is referred to in the Results section.**

**2.** Purification of mucins used 5 × 10^10^ parasites, but which forms were used is not explicit? Presumably they were epimastigotes though, in which case it should be clarified what is known about gp82 and gp35/50 expression in epimastigotes. This would help with the interpretation of figure 2.

**R.** **We incorporated in the revised manuscript the information that gp35/50 mucins were purified from cultures containg mostly epimastigotes, which do not express gp82 (Teixeira and Yoshida, 1986), and that the glycosylphosphatidyl anchor and** the O-linked sugar chains are essentially the same in epimastigotes and MT, **as reported by Serrano et al. (1995).**

**3.**  Perhaps a naïve biochemistry question, but how can we be sure that the F3 fraction contains purely mucins? The authors state “We followed the protocol used previously to purify mucins from different T. cruzi strains [32=Maeda et al 2011].” However, the Maeda paper references the protocol further back to a review article (Acosta-Serrano et al 2001) that does provide any more details about the protocol or its validation.

**R.** **As a matter of fact, the protocol of mucin purification was based on the procedure described by Serrano et al. (1995). The reference Maeda et al. (2012) was replaced by Serrano et al. (1995). According to this protocol, pure mucins from epimastigotes and MT are obtained in fractions F2 and F3, suitable for structure determination.**

4.  Immunofluorescence images (Figures 1,2,3 etc) are all reported qualitatively. The phenotypes look convincing enough so quantitative image analysis would probably be overkill, but I think the methods and or figure legends should indicate how many fields of view were checked for consistency with these representative images.

**R. An average of 10 fields were checked for consistency with the representative images shown in Results. This information was incorporated in the Methods section.**

5.  Why/how did they decide to focus specifically on Annexin A2? I think a line of explanation is needed at the start of the section at Line 281.

**R. The gp35/50-mediated invasion of G strain MT is closely associated with target cell actin cytoskeleton, and is inhibited upon treatment with F-actin-disrupting drugs, as reported (Ferreira et al., 2006). We decided to focus specifically on annexin A2 because of its importance as actin nucleator in the vicinity of cellular membranes (Hayes et al., 2009, Grieve et al., 2012). A line of explanation was added accordingly.**

6.  Figure 3C and related text – should clarify if these are uninfected or infected cells.

**R. Cells shown in Figure 3C (Figure 4C in revised manuscript) were not infected. This was made clear in the figure legend and in the text.**

7.      Line 325, epitelial -> epitelial

**R. Corrected.**

8.      Figure 6, the specificity of the sucrose pre-treatment to clathrin-mediated endocytosis seems questionable, so I think the data on parasite internalisation in this figure should be supported by the clathrin and mucin immunofluorescence assays as per figure 5. This should reveal/confirm the extent to which the sucrose has affected calthrin-parasite/mucin co-localisation.

**R.** **The suggested immunofluorescence assays were performed, and the result showing reduced clathrin-MT/mucin co-localization in sucrose-treated cells, as compared to untreated controls, is presented in Figure 7C and Figure 8.**

9.      Line 332 “G strain MT invasion does not require gp82”. I’m not sure that the experiment allows such a definitive statement to be made, I think they would need to delete the gene to prove it is not required. It would be useful to know whether antibody concentration is a factor (is the augmentation dose-dependent?), the amount used is not stated. Also, according to the reference #30 (Teixeira et al 1986), the Mab 3F6 binds proteins of MW 82kDa and 75kDa, so how can specific inferences be made about gp82? Discussion relating to these data could be re-phrased for the same

reasons.

**R. We agree that the *gp82* gene need to be deleted to prove that the protein is not required for invasion, a task that has not been attempted because gp82 is encoded by a multigene family whose members are distributed in several chromosomes (Araya et al, 1994). The assay with antibody was performed with ascitic fluid. To determine the effect of concentration, as suggested, mAb 3F6 was purified and used in an addtional assay. Gp82 contains N-linked oligosaccharide chains. Upon treatment of MT with tunicamycin, an inhibitor of N-glycosylation, the 82 kDa protein is converted into a protein of about 70 kDa. The 75 kDa protein may correspond to the non-glycosylated protein not fully exposed on the parasite surface. Discussion on the topic was reformulated.**

10.     Line 383, date -> data

**R. Corrected.**

11.     I think the discussion would benefit from some comments on the importance of the results in the context of the broader diversity of T. cruzi, the potential difference between metacyclics and blood/tissue culture trypos and the cell tropism in vivo (how representative are HeLa cells?)

**R. We have added in the discussion the following paragraph, containing comments on the questions raised by the reviewer. “Gp35/50 mucins, which are highly glycosylated and resistant to proteolytic degradation (Acosta Serrano et al.., 1995, Mortara et al., 1992), play an important role in oral *T. cruzi* infection, which is currently a frequent mode of transmission. Studies in mice have shown that metacyclic forms invade the gastric mucosal epithelia as a portal of entry for systemic infection (Hoft et al., 1996, Yoshida, 2008). The resistance of MT of different *T. cruzi* strains against peptic digestion is presumably conferred by gp35/50 mucins. When recovered from the mouse stomach 1 h after oral inoculation, the infectivity of MT of different strains towards HeLa cells was fully preserved, similarly to the MT ability to invade cells upon treatment with pepsin at acidic pH (Covarrubias et al., 2007). Differently from MT, bloodform trypomastigotes cannot efficiently initiate mucosal infection after an oral challenge (Hoft, 1996) and this may be associated with the partial susceptibility of mammalian stage trypomastigotes to some proteases (Acosta et al., 2001)”.**

**Reviewer 2**

Specific comments:

-       In the M & M section, authors describe the purification of T. cruzi mucin-like molecules. In line 142, after “A total of 5 x 10^10^10 parasites”, I suggest to add the strains of parasites used (G and CL strains).

**R. The parasites strains were added, as suggested.**

-       In this point (line 157-158), authors indicate that “fractions were analyzed by silver staining of SDS-PAGE gels, as well as by immunoblotting using the available monoclonal antibodies”. Were these analyses used to identify different mucins present in these fractions (gp35/50, gp90 and gp82). If it is possible, I suggest, to add this characterization to the results and the proportion of specific mucins in MUC-G and MUC-CL. In case this characterization has been published previously, I suggest incorporating a reference.

**R. A figure (Figure 1) was incorporated, showing the gp35/50 mucins purified from G and CL strains analyzed by silver staining and Schiff staining, as well as by western blotting using specific monoclonal antibodies. The purified fraction does not contain either gp82 or gp90, which are glycoproteins structurally distinct from gp35/50 mucins. Gp82 and gp90 contain N-linked oligosaccharides, wheras gp35/50 mucins are highly highly glycosylated proteins rich in threonine, with a unique type of glycosylation consisting of several sialylated *O*-glycans linked to the protein backbone via *N*-acetylglucosamine residues. This information was incorporated in the revised manuscript.**

-       In some sections of results, authors point out a purified gp35/50 (line 307). It is not completely clear if this purified gp35/50 is a synonym of MUC-G. Please clarify.

**R. Purified gp35/50 is a synonym of MUC-G. We substituted purified gp35/50 for MUC-G.**

-       Figure 8. The schematic representation of possible mechanisms of host cell invasion by T. cruzi strains G and CL, is a good figure that summarizes the main results. However, some important information highlighted here is presented in supplementary material. In this sense, I suggest incorporating S2 and S4 into the article’s figures.

**R**. **A new figure (Figure 2A) was generated, containing data from previous Figures 1A and S2. Figure S4 was placed as Figure 9D.**

-       The abstract, summary and results are focused mainly in gp35/50. Therefore, I recommend replacing “mucin molecules” by gp35/50 in the title.

**R**. **The title was changed to “Gp35/50 mucin molecules....”**

-       The resolution of figures should be improved.

**The resolution of figures was increased. Figures files were uploaded to the**

**Preflight Analysis and Conversion Engine (PACE) digital diagnostic tool, and they**

**meet PLOS requirements.**

- The study was approved by a Committee on Ethics of Animals despite not incorporating in vivo assays.

**R. The study was submitted to approval by the Committee on Ethics of Animal Experimentation because along the study *T. cruzi* strains were maintained alternately in mice and in culture medium.**

- An approved biosecurity certificate should be incorporated.

**R. Included, as recommended.**

Reviewer 3

he report is well written and easily followed, leading to confirmation of previous findings and further supporting knowledge of parasite's invasion strategies.
However, the observation of protein phosphorylation without involvement of the affected proteins together with the poor focus of the hypertonic medium strongly cloud the report. To fully support the involvement of clathrin-coated vesicles in parasite invasion, several assays with appropriate inhibitors must be performed.

**Additional experiments were performed to identify the protein tyrosine kinase involved in gp35/50-mediated G strain MT invasion. We tested the possibility that focal adhesion kinase (FAK) might be activated by MUC-G and MT. FAK is a cytoplasmic PTK with a molecular mass of 125 kDa, which regulates the F-actin dynamics (Parsons et al., 2000; Li et al., 2013).** **Both MUC-G and MT were found to activate FAK. In addition, it was found that the treatment of HeLa cells with specific FAK inhibitor decreased MT internalization. These data were incorported as Figures 5B and 5C. To fully support the involvement of clathrin-coated vesicles in MT invasion, additional assays were performed. Immunofluorescence analysis of untreated and sucrose-treated cells, upon incubation with MT, showed reduced clathrin-MT/mucin co-localization in sucrose-treated cells, as compared to untreated controls. The result was incorported as Figure 7C and Figure 8. Hela cells were also subjectd to several procedures, such as treatment with chlorpromazine, intracelular K^+^ depletion or cytoplasmic acidification, reported to affect clathrin-coated pits and endocytosis (Larkin et al., 1983, Cosson et al., 1989, Barrias et al., 2019). The susceptibillity to MT invasion of these cells was significantly reduced, as compared to untreated cells. This result was added as Figure S4.**
